# Best of UEG Week 2024

**DOI:** 10.1002/ueg2.12746

**Published:** 2024-12-24

**Authors:** Anna Burelli, Anthea Pisani, Zsa Zsa R. M. Weerts, Irene Marafini, Ana Dugic

**Affiliations:** ^1^ General Surgery Unit IRCCS Sacro Cuore Don Calabria Hospital Negrar di Valpolicella, Verona Italy; ^2^ Division of Gastroenterology Department of Medicine Mater Dei Hospital Msida Malta; ^3^ Department of Gastroenterology and Hepatology Maastricht University Medical Center+ Maastricht Netherlands; ^4^ Department of Systems Medicine University of Rome Tor Vergata Rome Italy; ^5^ Gastroenterology Unit Department of Medical Science Tor Vergata University Hospital Rome Italy; ^6^ Department of Internal Medicine IV Heidelberg University Hospital Heidelberg Germany

Dearest Readers,

Another terrific UEG Week (UEGW) has passed and please allow us to vividly recount it through the eyes of three delegates: ‘U’, ‘E’ and ‘G’, our three protagonists who wandered enthusiastically through the congress venue and attended lots of events. U is a colorectal surgeon with a strong interest in IBD, E a resident hoping to become an advanced endoscopist and G a researcher focusing on hepatobiliary diseases.

They all arrived in Vienna with great expectations and the UEGW was able to, once again, astound them with more than 11K registrants (11% virtual) coming from 114 countries and from all continents. The Post Graduate Teaching programme (PGT) registered nearly 5K attendees. Italy was the top‐attending country, followed by Germany, the United Kingdom, and the United States. The Research Committee did a mammoth job selecting 495 Faculties, 2477 abstracts and 2200 posters.

U arrived early and went straight to the first session of the PGT. This is a 3‐year curriculum, with 2024 covering year 1. Two to four topics for each area (colorectal, endoscopy and imaging, IBD, liver, oesophagus, nutrition, pancreas and biliary tract, stomach, therapy update) were presented with a focus on everyday clinical practice. This year's opening session covered metabolic dysfunction, shedding an important insight on the interaction between alcohol use and cardiometabolic risk factors in steatotic liver disease [1]. It was there where U tried the new AI‐powered translation service and was excited by the remarkable real‐time translations provided in Dutch during live sessions.

In the meantime, E was determined to gain some new practical skills and looked into the Hands‐on programme including the Gastroenterologist‐Surgeon Collaboration and Endoanal Ultrasound (US) course, finally settling on the abdominal US course on Saturday and a pre‐registered EMR session on Sunday. The US course offered an excellent mixture of lectures and hands‐on practice, while the endoscopy session provided a chance to practice in a simulated environment. E also sneaked a peek into the new vibrant ‘Emergency Training in Endoscopy’ session where participants were actively resuscitating an unwell patient.

G, a first‐time delegate, was overwhelmed by the myriad of things to do and went to the myUEG Community Area looking for help. There, valuable tips were provided at the *How to make the most of your first UEG Week experience* session. The myUEG Community Area is the beating heart of the UEGW, where delegates can meet and get to know (future) collaborators, mentors, editors, awardees and participants with similar interests. The *Learn from leaders* activities are tailored for early career delegates and include *Young GI mentoring, How to review a scientific manuscript, How to deal with poor outcomes, How to lead a global network of researchers* and many more. A vibrant session hosted by the *UEG Journal* (*@UEGJournal & X*) illustrated in Figure 1 focused on boosting professional influence through social media [2, 3]. It brought together young GIs from around the globe and featured well‐known social media experts sparking vibrant engagement among participants. Aline Charabaty (@DCharabaty), Keith Siau (@drkeithsiau) and Beatriz Gros (@Bealoquebea) illustrated how social media facilitates many work life aspects as career advancements, multidisciplinary connections, research collaborations and interpersonal relations and how weekly appointments as #MondayNightIBD represent an incredible learning opportunity that engages lively discussions.

**FIGURE 1:**
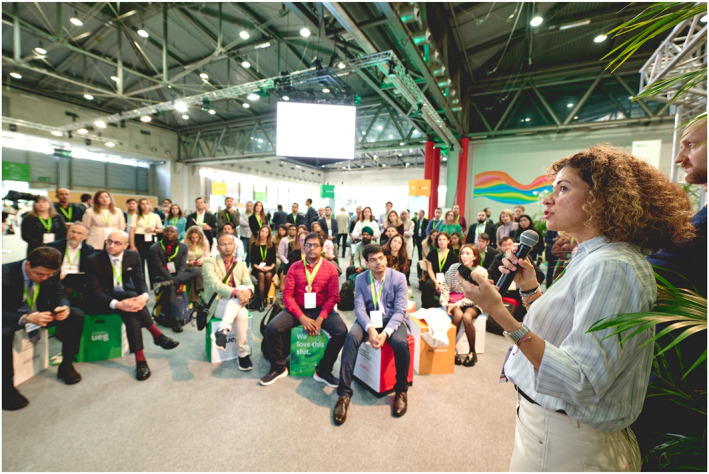
*@UEGJournal & X* session at the myUEG Community Area.

As the first day of the congress wrapped up, our protagonists received an invitation from their peers to join the ‘Let's Meet’ party for young GI delegates. The party featured fun games where attendees found pairs using wristbands with terms from the gastroenterology field. They enthusiastically embraced this chance to dive into an energetic scene, forging new friendships!

The official congress kick‐off was the opening plenary on Sunday afternoon. The participation was massive. The innovative lecture by Marlies Schijven *Serious Gaming in GI‐disease: Playfully preparing Generation Z* kept all eyes wide open (Figure 2). It explored generational differences from baby boomers (1945–1960), to generation X (1961–1980), Y (1981–1995) and Z (born after 1995) in leisure and working habits and consequently in learning. If baby boomers and generation X learnt with chalk boards, generation Y became tech‐savvy and now generation Z, who spends more than 25% of its time on gaming, needs a playful environment to learn. A paradigm shift in learning is needed if we want to provide society with well‐trained gastroenterologists and surgeons as there is a thin optimal experience line between the anxiety area (too stressful environment) and the boredom area (not engaged).

**FIGURE 2:**
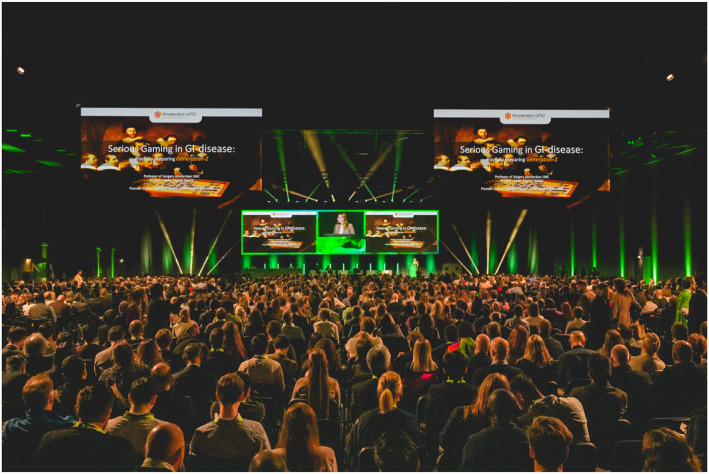
*Serious Gaming in GI‐disease: Playfully preparing Generation Z* plenary session.

The plenary went on with the five top abstracts award ceremony [4]:

1. SIRNA/CS‐PLGA nanoparticle system targeting knockout of intestinal SOAT2 reduced intestinal lipid uptake and alleviate obesity—Wentao Shao (China).
2. RXC008: first‐in‐class gastrointestinal‐targeted potent pan‐rock inhibitor for treatment of fibrostenotic crohn's disease—Elaine Kilgour (United Kingdom).
3. Optical PD‐L1 imaging using ultrasound‐guided quantitative fluorescence molecular endoscopy combined with durvalumab‐680LT in locally advanced oesophageal cancer patients—Anne M. van der Waaij (Netherlands).
4. Identifying putative genomic biomarkers for risk stratification in Barrett's oesophagus patients with normal histological features—Pim Stougie (Netherlands).
5. Crohn's disease and ulcerative colitis exhibit pre‐diagnostic antibody signatures with shared and divergent changes towards disease onset—Arno R. Bourgonje (Netherlands).

The latter work was presented during the plenary and ignited a spirited discussion on pre‐clinical IBD changes. Thereafter, it was the turn of the UEG Research Prize 2024 awardee Enrique De Madaria who presented the premises of the WATERLAND study [5] and held an inspiring lecture on the five elements for a successful research career:

1. Fire: have passion and enjoy the voyage.
2. Earth: learn methodology from the beginning.
3. Air: set your sails towards changing clinical practice.
4. Water: be constant as the waves in the sea.
5. Ether: have good values, think about the benefit of patients, be a good person, be generous with your collaborators.

The presidential address by Mathias Löhr underlined the importance of being a solid scientific community aiming *onwards and upwards* as per the presidency's motto [6]. Multidisciplinarity is the strength of this community counting > 30,000 myUEG associates whose personal and work connections are boosted by a unique cyber‐secured app: MyConnect. Finally, the UEGW 2025 venue was presented: Berlin 4–7 October.

The plenary lecture on gut hormone co‐agonists for the treatment of metabolic syndrome revealed the deep faith of Matthias Tschöp in double and triple agonists in curing obesity with a domino positive effect on diabetes, cardiovascular diseases, sleep apnoea, nicotine, alcohol addiction, and, possibly, cancers and dementia. The session concluded with Jeanine van Hooft honouring former UEG President Paul Fockens for his distinguished career and presenting him with the UEG Lifetime Achievement Award 2024 [7].

Congress sessions took off, from ‘*Therapy updates*’ to ‘*What's New In… in 2024?*’ passing by ‘*From guidelines to clinical practice*’ and the famous ‘*Mistakes In…*’ sessions. G attended the one on hepatobiliary and pancreas issues and made sure to pick up a printed copy of the ‘Mistakes in…’ collection to use in clinic the rest of the year. Everyone was impressed with the huge variety of formats. ‘*Clinical Case Discussion*’ and ‘*Never Waste a Good Disaster*’ sessions were definitely appreciated, while presented cases were easy to relate to and where one could learn from the experiences of others. Inevitably many sessions occurred simultaneously, but they were all video‐recorded and available thereafter on the on‐demand congress platform. For those who look for a quick recap, a special edition of the UEG Talks Podcast dedicated to the best of UEGW 2024 (endoscopy, IBD, Bench to bedside, HPB, Oncology for Gastroenterologist, Nursing) has been released after the congress.

At the end of the day, U, E and G grasped the opportunity to see what original research their peers were performing by visiting the Science Lounge. Looking at the e‐posters, they took the chance to network with others and exchange ideas. They listened to various moderated poster sessions and participated in the Poster Rally, a quiz based on the posters, with PGT 2025 registrations up for grabs.

Historically the UEG Week has always served as a great catalyst for in‐person networking, nurturing lifelong friendships and collaborations that span all career levels among GI professionals. Indeed, on Sunday, the congress hosted the Women in GI networking reception. This inspiring gathering brought together women in gastroenterology, allowing the youngers to connect with fellow professionals to whom they have looked up to for years, sharing insights, and engaging in discussions about mentorship, leadership, and challenges women face every day in this field.

On Monday, the UEG Night at the Kaiser Wiesn in Vienna's Prater Park was an unforgettable celebration, brimming with energy and camaraderie (Figure 3). As U, E and G arrived at the beautifully decorated big top, the festive atmosphere was palpable, with laughter and lively conversations filling the air. Embracing the spirit of Oktoberfest, U wore a stunning dirndl, while E and G looked sharp in their traditional lederhosen. The evening featured vibrant performances from an excellent band and delicious Austrian traditional cuisine, keeping everyone in high spirits. As the night unfolded, it became evident that this event was not just a highlight of UEGW but a true testament to the strength and unity of the myUEG Community.

**FIGURE 3:**
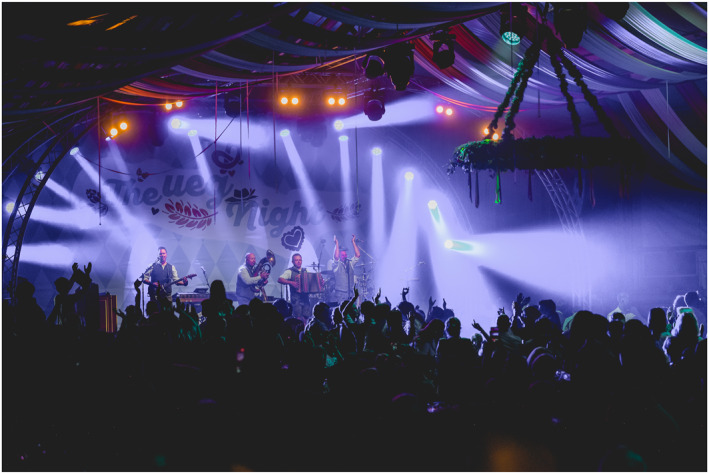
The UEG night.

On the last day of the congress, our protagonists, though still tired after the night, woke up bright and early to make the most of their last day. E went directly to the ESGE Live Endoscopy sessions, eager to spend the day observing the experts from Amsterdam University Medical Center performing procedures such as POEM, EMR, ESD and hepatobiliary procedures. The calm, professional way the experts handled complex procedures was fascinating and a few tips and tricks were to learn.

As the crowd leaves, our three friends realise that the success of UEGW is enabled by the people who make it possible: the UEG headquarters employees, committee chairs and members, and volunteers who work behind the scenes, and whose commitment not only enhances the experience for all attendees but also exemplifies the spirit of community that defines UEG.

Before travelling home, our protagonists meet up one last time to take a memorable photo at the Magic Mirror. Over the past 4 days, they have gained more than scientific knowledge, practical skills and CME points. They have made new connections and brimmed with new ideas and enthusiasm. They could not help to think of next year, UEGW in Berlin. They couldn't help but wonder: ‘Will the pretzels taste as good under the shadow of *Brandenburger Tor*?’

## Conflicts of Interest

The authors declare no conflicts of interest.

## Data Availability

The authors have nothing to report.
